# Inflammation mediates the relationship between obesity and retinal vascular calibre in 11-12 year-olds children and mid-life adults

**DOI:** 10.1038/s41598-020-61801-w

**Published:** 2020-03-19

**Authors:** Mengjiao Liu, Kate Lycett, Margarita Moreno-Betancur, Tien Yin Wong, Mingguang He, Richard Saffery, Markus Juonala, Jessica A. Kerr, Melissa Wake, David P. Burgner

**Affiliations:** 1 The University of Melbourne, Melbourne, VIC Australia; 2 Murdoch Children’s Research Institute, Melbourne, VIC Australia; 3 Centre for Social & Early Emotional Development, Deakin University, Melbourne, VIC Australia; 4 Department of Ophthalmic Epidemiology, Centre for Eye Research Australia, The University of Melbourne, Melbourne, Australia; 5 Singapore Eye Research Institute, Singapore National Eye Center, Singapore, Singapore; 6 State Key Laboratory of Ophthalmology, Zhongshan Ophthalmic Center, Sun Yat-Sen University, Guangzhou, China; 7 Department of Medicine, University of Turku, Turku, Finland; 8 Division of Medicine, Turku University Hospital, Turku, Finland; 9 Department of Paediatrics, Monash University, Melbourne, Australia; 10 Infectious Diseases, Royal Children’s Hospital, Melbourne, Australia

**Keywords:** Biomarkers, Chronic inflammation

## Abstract

Obesity predicts adverse microvasculature from childhood, potentially via inflammatory pathways. We investigated whether inflammation mediates associations between obesity and microvascular parameters. In 1054 children (mean age 11 years) and 1147 adults (44 years) from a cross-sectional study, we measured BMI (z-scores for children) and WHtR, Glycoprotein acetyls (GlycA), an inflammatory marker, and retinal arteriolar and venular calibre. Causal mediation analysis methods decomposed a “total effect” into “direct” and “indirect” components via a mediator, considering continuous and categorical measures and adjusting for potential confounders. Compared to normal-weight BMI children, those with overweight or obesity had narrower arteriolar calibre (total effects −0.21 to −0.12 standard deviation (SD)): direct (not mediated via GlycA) effects were similar. Children with overweight or obesity had 0.25 to 0.35 SD wider venular calibre, of which 19 to 25% was mediated via GlycA. In adults, those with obesity had 0.07 SD greater venular calibre, which was completely mediated by GlycA (indirect effect: 0.07 SD, 95% CI −0.01 to 0.16). Similar findings were obtained with other obesity measures. Inflammation mediated associations between obesity and retinal venules, but not arterioles from mid-childhood, with higher mediation effects observed in adults. Interventions targeting inflammatory pathways may help mitigate adverse impacts of obesity on the microvasculature.

## Introduction

Obesity is a leading risk factor for cardiovascular disease (CVD), with adverse effects on the vasculature, including the microcirculation^1,2^. Obesity is associated with adverse microvascular parameters from early childhood, but underlying mechanisms remain unclear^3,4^. Inflammation is suggested to play a key role^5^.

Retinal microvasculature, which has been shown to mirror changes in other vascular beds, is a surrogate marker for systemic microvasculature^6^. Investigation of the retinal microvasculature is now feasible in population-based cohort studies by retinal imaging technique^7^, which is well-tolerated and reproducible from early childhood onwards^8^. Population-based studies have shown that increasing body mass index (BMI) and waist-to-height ratio (WHtR), two commonly used measures related to obesity, are associated with narrower retinal arterioles and wider venules in both children and adults^5,9–11^.

Inflammatory markers, such as high sensitivity C-reactive protein (hsCRP) and white blood cell count, have been associated with adverse retinal microvascular variation in adults, most consistently with venular rather than arteriolar calibre^3,12,13^. In children, acute phase reactants, such as hsCRP, are less informative about chronic inflammation. A novel biomarker, glycoprotein acetyls (GlycA), is suggested to be more indicative of chronic and cumulative inflammation in children and adults^14,15^. Few studies of the relationship between inflammation and the microvasculature have considered the role of obesity, a key driver of chronic inflammation^12,13^.

**Figure 1:**
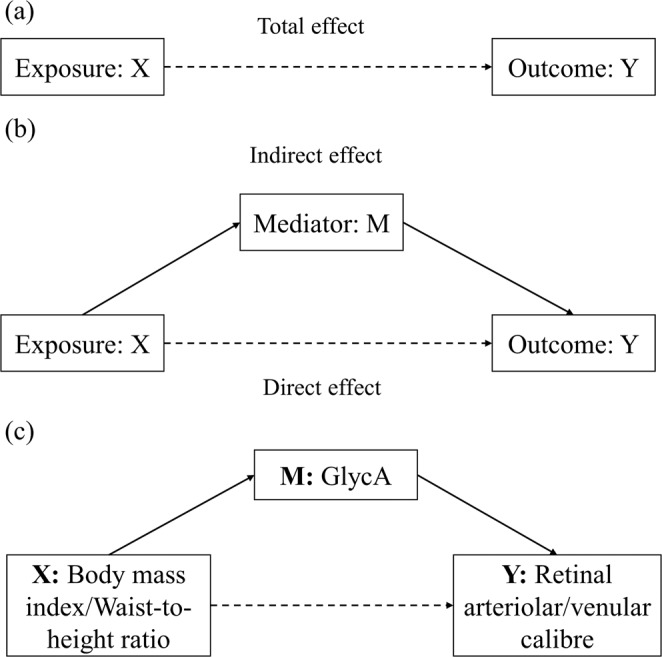
Causal mediation framework. (**a**) The effect of exposure X on outcome Y without taking into account mediator M is known as the “total effect”. (**b**) The effect of X on Y not via M is known as the “direct effect”, and the effect through M is the “indirect effect”. (**c**) X, M, Y are body mass index /waist-to-height ratio, glycoprotein acetyls (GlycA) and retinal arteriolar/venular calibre in the current study. Confounders included in the analysis were not shown in the figure.

The question of whether the association between obesity and retinal microvasculature is explained by inflammatory pathways can be addressed within a causal mediation framework (Fig. 1a,b). Theoretically, this framework enables estimation of the extent to which the total causal effect of an exposure on an outcome occurs via an intermediate variable (“mediator”), by decomposing the total effect into an “indirect” effect through the mediator and a “direct” effect through other pathways.

While the “total (causal) effect” of obesity on any outcome is ill-defined, it is relevant to examine the extent to which obesity-related disparities in retinal microvascular parameters would be reduced, if the distribution of inflammatory markers in those with obesity were reduced to levels observed in the non-obese^16^, thereby representing potential “interventional effects”^17,18^. Using this approach, we examined whether the inflammatory biomarker GlycA mediates the association between measures relating to obesity (BMI and WHtR) and retinal microvasculature (Fig. 1c), in 11–12 year olds and in mid-life adults. The child and adult sample can provide information regarding associations across the life course. We hypothesise that the association between obesity and retinal vascular calibre and the mediation effect of inflammation will be stronger in adults than in children.

## Results

### Sample characteristics

The study flow from wave 1 of LSAC onwards is shown in the Supplementary Figure. Of the 1874 participating families, 1054 children (mean age 11.4 years) and 1147 adults (their parents, mean age 43.8 years) had data on all measures. Table 1 shows the characteristics of the analytic sample. Over a quarter of children were in the overweight (14.4%) and obese (8.6%) categories, and 8.6% had WHtR ≥0.5. In adults, 62.2% were in the overweight and obese categories, and 53.9% had WHtR ≥0.5.

**Table 1:** Sample characteristics.

Characteristics	Children (n = 1054)	Adults (n = 1147)
	Means (SD^a^) or %	Means (SD^a^) or %
Age (years)	11.4 (0.5)	43.8 (5.1)
Sex (% female)	52.0	86.7
Socioeconomic position	0.3 (0.9)	0.3 (1.0)
Body mass index z-score^b^	0.3 (1.0)	
Body mass index (kg/m^2^)		27.9 (6.1)
**Current BMI status (%)**		
Normal	77.0	37.8
Overweight	14.4	32.9
Obese	8.6	29.3
Waist-to-height ratio (WHtR)	0.4 (0.1)	0.5 (0.1)
**Current WHtR status (%)**		
<0.5	91.4	46.1
≥0.5	8.6	53.9
Retinal arteriolar calibre (µm)	159.0 (11.9)	151.1 (13.9)
Retinal venular calibre (µm)	230.9 (16.5)	219.0 (18.6)
Glycoprotein Acetyls (mmol/L)	1.0 (0.1)	1.0 (0.2)

^a.^Standard deviation; ^b^Body mass index was transformed to z-scores using the Centres for Disease Control and Prevention (US) growth charts.

### Interrelationships between key variables

Table 2 shows results from multivariable linear regression models, adjusted for age, sex and SEP. BMI and WHtR were moderately associated with GlycA, with stronger evidence for associations in adults than children. For example, in children and adults, one SD greater in BMI were associated with 0.39 SD (95% CI 0.33 to 0.45) and 0.47 SD (95% CI 0.42 to 0.52) higher GlycA scores, respectively.

**Table 2:** Inter-relationships of key variables, assessed via multivariable linear regression models, per SD unit higher in the exposure (adjusted for age, sex and SEP).

Models (the exposure and outcome)	Children		Adults	
	Effect size (95% CI)	*p*	Effect size (95% CI)	*p*
**BMI/WHtR and proposed inflammatory mediator**				
BMI and GlycA	0.39 (0.33, 0.45)	<0.001	0.47 (0.42, 0.52)	<0.001
WHtR and GlycA	0.44 (0.38, 0.49)	<0.001	0.53 (0.48, 0.57)	<0.001
**BMI/WHtR and retinal vascular calibre outcomes**				
BMI and arteriolar calibre	−0.12 (−0.19, −0.06)	<0.001	−0.16 (−0.21, −0.08)	<0.001
BMI and venular calibre	0.03 (−0.04, 0.09)	0.33	0.02 (−0.04, 0.07)	0.58
WHtR and arteriolar calibre	−0.10 (−0.16, −0.06)	<0.001	−0.16 (−0.22, −0.10)	<0.001
WHtR and venular calibre	0.05 (−0.01, 0.12)	0.08	0.03 (−0.03, 0.08)	0.36
**Proposed inflammatory mediator and retinal vascular calibre outcomes**				
GlycA and arteriolar calibre	−0.02 (−0.08, 0.04)	0.52	−0.09 (−0.15, −0.03)	<0.01
GlycA and venular calibre	0.09 (0.03, 0.15)	<0.01	0.06 (0.00, 0.12)	0.03
Proposed inflammatory mediator and retinal vascular calibre outcomes*				
GlycA and arteriolar calibre	0.03 (−0.03, 0.09)	0.41	−0.02 (−0.08, 0.05)	0.59
GlycA and venular calibre	0.09 (0.03, 0.16)	<0.01	0.07 (0.01, 0.14)	0.03

Abbreviations: 95%CI, 95% confidence interval; BMI, body mass index; WHtR, waist-to-height ratio; GlycA, glycoprotein acetyls.

*The model estimates were further adjusted for body mass index as a confounding factor.

Higher BMI and WHtR showed small associations with narrower arteriolar calibre, with effects slightly larger in adults than children. In contrast, the associations of BMI with venular calibre were weak for both children and adults (0.03 (95% CI −0.04 to 0.09), 0.02 (95% CI −0.04 to 0.07), respectively). Similar findings were observed for WHtR and venular calibre.

Higher GlycA had small associations with narrower arteriolar calibre in adults but not children. In adults, one SD higher GlycA was associated with −0.09 SD (95% CI −0.15 to −0.03) narrower arteriolar calibre. Further adjustment for BMI fully attenuated this association in adults. The association between higher GlycA and wider venular calibre in children was less evident in adults, and BMI did not appear to confound this association.

### Does GlycA mediate the association of obesity with retinal vascular calibre?

**Figure 2:**
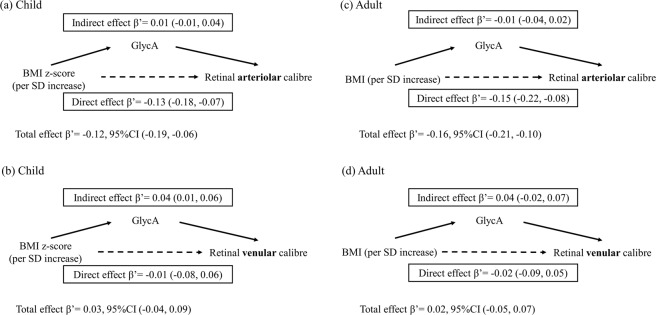
Mediation effects of GlycA on associations of body mass index (z-scores for children, standardised scores for adults) with standardised retinal venular and arteriolar calibre. Footnotes: β’ represents effect estimates for direct, indirect, and total effect derived from causal mediation analysis. SDs of retinal arteriolar and venular calibre in children are 11.9 µm and 16.5 µm, and SDs of BMI, retinal arteriolar and venular calibre in adults are 6.1 kg/m^2^, 13.9 µm and 18.6 µm. Abbreviations: 95%CI, 95% confidence interval; SD, standard deviation; BMI, body mass index; GlycA, glycoprotein acetyls.

**Figure 3:**
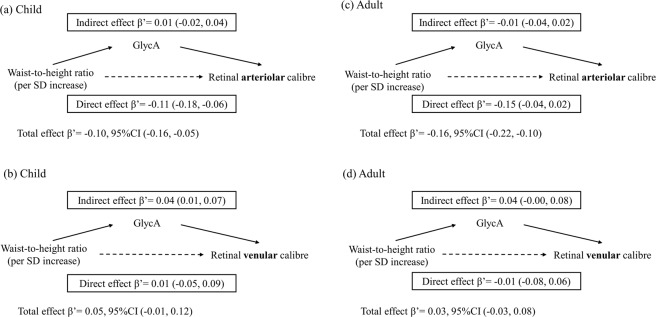
Mediation effects of GlycA on associations of standardised waist-to-height ratio with standardised retinal venular and arteriolar calibre. Footnotes: β’ represents effect estimates for direct, indirect, and total effect derived from causal mediation analysis. SDs of waist-to-height ratio, retinal arteriolar and venular calibre in children are 0.1, 11.9 µm and 16.5 µm, and SDs of waist-to-height ratio, retinal arteriolar and venular calibre in adults are 0.1, 13.9 µm and 18.6 µm. Abbreviations: 95%CI, 95% confidence interval; SD, standard deviation; WHtR, waist-to-height ratio; GlycA, glycoprotein acetyls.

The results of the causal mediation analysis when exposures were considered as continuous variables are presented in Figs. 2 and 3. Although BMI had a small “total effect” on retinal arteriolar calibre (Fig. 2a,c), the direct effects, not mediated via GlycA, were similar to the total effects in children and adults. In contrast, BMI had a smaller effect on venular calibre for children and adults (Fig. 2b,d), with some evidence of mediation through GlycA in both groups. Direct effects, not involving GlycA, were in the opposite direction for children (−0.01 SD, 95% CI −0.08 to 0.06) and adults (−0.02 SD, 95% CI −0.01 to 0.05). The results were similar when the exposure was WHtR (Fig. 3).

When considering exposures as categorical variables, compared to those with normal weight, those with overweight or obesity - whether either measured by BMI or WHtR - had narrower arteriolar calibre, but again the direct effects not mediated via GlycA were similar to the total effects (Table 3).

**Table 3:** Direct and indirect effects of overweight and obesity on retinal vascular calibre through GlycA; estimates adjusted for age, sex and SEP.

Obesity category	Children				Adults			
	Retinal arteriolar calibre		Retinal venular calibre		Retinal arteriolar calibre		Retinal venular calibre	
	β’ (95% CI)	*p*	β’ (95% CI)	*p*	β’ (95% CI)	*p*	β’ (95% CI)	*p*
**BMI: Overweight vs normal (ref)**								
Indirect effect	0.00 (−0.05, 0.05)	0.93	0.05 (0.00, 0.10)	0.05	−0.01 (−0.04, 0.03)	0.71	0.03 (−0.00, 0.06)	0.08
Direct effect	−0.11 (−0.32, 0.10)	0.30	0.20 (0.01, 0.40)	0.04	−0.23 (−0.36, −0.08)	<0.01	−0.01 (−0.17, 0.13)	0.81
Total effect	−0.11 (−0.32, 0.10)	0.33	0.25 (0.06, 0.45)	0.01	−0.24 (−0.36, −0.10)	<0.01	0.02 (−0.13, 0.16)	0.85
Proportion mediated by GlycA	−0.01 (−1.98, 2.76)	0.96	0.19 (−0.00, 0.78)	0.06	0.03 (−0.13, 0.23)	0.71	1.92 (−5.12, 7.21)	0.88
**BMI: Obese vs normal (ref)**								
Indirect effect	0.00 (−0.08, 0.09)	0.88	0.09 (0.01, 0.18)	0.04	−0.01 (−0.04, 0.03)	0.71	0.07 (−0.01, 0.16)	0.08
Direct effect	−0.14 (−0.42, 0.14)	0.32	0.26 (−0.04, 0.55)	0.09	−0.23 (−0.37, −0.09)	<0.01	0.00 (−0.18, 0.16)	0.95
Total effect	−0.13 (−0.41, 0.13)	0.32	0.35 (0.07, 0.64)	0.02	−0.24 (−0.37, −0.10)	<0.01	0.07 (−0.08, 0.21)	0.34
Proportion mediated by GlycA	−0.02 (−2.40, 2.64)	0.92	0.25 (−0.00, 1.25)	0.06	0.03 (−0.13, 0.23)	0.71	1.00 (−6.86, 10.23)	0.39
**WHtR: ≥0.5 vs** <**0.5 (ref)**								
Indirect effect	−0.00 (−0.09, 0.08)	0.98	0.10 (0.02, 0.18)	0.01	−0.03 (−0.10, 0.03)	0.27	0.05 (−0.02, 0.12)	0.12
Direct effect	−0.18 (−0.41, 0.03)	0.11	0.13 (−0.08, 0.38)	0.22	−0.22 (−0.35, −0.09)	<0.001	0.02 (−0.12, 0.15)	0.76
Total effect	−0.18 (−0.41, 0.04)	0.10	0.23 (0.03, 0.46)	0.02	−0.25 (−0.37, −0.14)	<0.001	0.07 (−0.04, 0.19)	0.21
Proportion mediated by GlycA	0.01 (−1.29, 0.92)	0.98	0.41 (0.04, 1.99)	0.03	0.14 (−0.09, 0.46)	0.27	0.69 (−5.69, 7.24)	0.30

β’ represents effect estimates for direct, indirect, and total effect derived from causal mediation analysis, compared to the reference group.

The proportion of the total effect mediated by GlycA was calculated as the ratio of the indirect effect to the total effect.

Abbreviations: 95%CI, 95% confidence interval; BMI, body mass index; WHtR, waist-to-height ratio; GlycA, glycoprotein acetyls.

Children with overweight and obesity had 0.25 SD and 0.35 SD wider venular calibre (respectively) compared to those with normal-weight BMI, of which 19 to 25% was mediated via GlycA (indirect effect: overweight 0.05 SD, 95% CI 0.0 to 0.10; obese 0.09 SD, 95% CI 0.01 to 0.18). In adults, retinal venular calibre was similar for normal weight and overweight, but adults with obesity had 0.07 SD greater venular calibre and this effect was completely mediated by GlycA (indirect effect: 0.07 SD, 95% CI −0.01 to 0.16). Similar mediation effects were seen in children and adults with WHtR ≥0.5 compared with those WHtR <0.5, with 41% to 69% of the association with venular calibre mediated by GlycA, respectively.

## Discussion

This is the first study to investigate whether the associations of obesity-related measures with microvascular parameters are mediated via inflammation. We showed that BMI and WHtR were associated both with GlycA and with adverse retinal vascular calibre (narrower arterioles and wider venules). Novel findings were that higher GlycA was associated with both wider venular calibre (in mid-childhood and mid-life adults) and narrower arteriolar calibre (in adults). Inflammation mediated associations between both measures related to obesity and retinal venular, but not arteriolar, calibre, with adults had higher mediation effects than children.

Our large, cross-generational cohort with standardised measurements using the same equipment and protocols may minimise potential measurement bias. The associations were investigated in children and adults, providing data from two ages in the life course. Similar results were observed for BMI and WHtR, which increases confidence in our findings, although these measures are highly correlated in children and adults (correlation coefficients 0.83 and 0.92 respectively). The use of GlycA, relatively novel in population-based studies^19^, may be advantageous given limitations of acute phase reactants in capturing chronic inflammation, especially in children^15^.

Potential limitations include the difficulty in interpreting mediated effects causally given the lack of a well-defined intervention on inflammation, so that our study can only suggest this as a potential intervention target. In addition, the cross-sectional and observational study design means there is potential for residual or unmeasured confounding. We assumed that there was no post-exposure confounding of mediator-outcome relationships that could arise through other potential mediating pathways. This assumption could be flawed; for example, higher LDL, another potential mediator, which may be increased in those with obesity and associate with narrower arteriolar and wider venular calibre directly^20^, or via the LDL-inflammation association to indirectly associate with poor retinal microvascular parameters^21^. However, in our additional analyses, LDL appears to confound the association with venular calibre only slightly in adults (Supplementary Table). Our participants were marginally more advantaged socioeconomically than the original nationally representative LSAC sample, so our conclusions may be less relevant to disadvantaged populations. Our analysis and findings were based on an existing cohort. Pre-specified analyses from future prospective studies are warranted to replicate the findings.

Retinal arteriolar and venular calibre showed different patterns of association with BMI and WHtR, in keeping with current evidence that they may be influenced by different factors^22^. Our study supports previous findings that inflammation is more consistently associated with retinal venular than arteriolar calibre in children and adults. The Generation R Study of children aged 6 years found that each SD higher CRP was associated with 0.10 SD (95% CI 0.06 to 0.14) wider venular calibre^3^. Similar findings are reported in mid-life and older adults and with other inflammatory markers, such as fibrinogen and white blood cells count^23–27^. GlycA is a novel composite inflammatory marker with greater stability and lower intra-individual variability than single acute phase inflammatory proteins, such as hsCRP^28^. In population studies, GlycA is a robust predictor of all-cause of mortality and CVD, even after adjusting for hsCRP^29–31^. In adolescents, GlycA is associated with obesity and prediabetes^32^. To the best of our knowledge, this is the first study to demonstrate that GlycA is associated with retinal venular calibre in children and adults and the magnitude of association is similar to previous findings.

We extended prior studies by exploring whether inflammation mediated the associations of obesity and retinal microvascular parameters. We found that the association of obesity with venular calibre was mediated by inflammation in both age groups. Data from both population-based studies and animal experiments support this interpretation. Yau *et al*. reported that in adolescents, low-grade inflammation partially mediates the relationship between cardiovascular fitness and more favourable retinal venular, but not arteriolar parameters^33^. In animal models, increased adiposity results in adipocyte dysfunction with increased pro-inflammatory cytokines and leukocyte activation, which may lead to the destruction of venular endothelium and consequently to wider retinal venular calibre^34^. In this study, the effect size in adults was small with wide confidence intervals, reflecting some lack of precision in our estimation of the degree of mediation by inflammation.

In contrast, although there is a small association between obesity and arteriolar calibre, the association between inflammation and arteriolar calibre was weak. Thus, the relationship of obesity and arteriolar calibre is unlikely to be mediated via inflammatory pathways, but by other non-inflammatory pathways. For example, obesity is a well-recognised risk factor for elevated blood pressure, a key determinant of retinal arteriolar calibre^35^. Structural adaptations of the endothelium and vessel wall in chronic hypertension may contribute to narrowing of the retinal arteriolar lumen^36^. Future studies could address the possible mediating role of blood pressure in this relationship.

Narrower retinal arterioles and wider retinal venules have been suggested as preclinical vascular phenotypes for CVD and in cohort studies have been associated with worse cardiovascular outcomes^37^. For example in older adults from the Atherosclerosis Risk in Communities study (n = 10470) with 16 years of follow-up, each SD increase in retinal venular and decrease in arteriolar calibre were associated with 18% and 14% higher risk of stroke, respectively (hazard ratio 1.18, 95% CI 1.07 to 1.31; 1.14, 95% CI 1.03 to 1.26)^38^. In our study, we observed a difference in retinal calibre in both children and adults with obesity: 0.35 SD and 0.07 SD wider venules, 0.13 SD and 0.24 SD narrower arterioles (respectively), compared to those with normal BMI. Obesity is a major risk factor of CVD, and children who have obesity are more likely to become adults with obesity^39^. Those with obesity have adverse microvascular parameters and may at risk of future CVD.

In addition, we found a large proportion of the association between obesity and retinal venular calibre was mediated via GlycA, 25–40% of the total effect in children and 70–100% in adults. This suggests that children have had less cumulative exposure to inflammation and that, with age, associations between obesity and adverse microvascular parameters may be increasingly mediated by inflammation. A large randomised control trial targeting interleukin-1ß (a prototypical inflammatory cytokine) has provided direct support for the ‘inflammatory hypothesis’ of CVD in humans^40^. Obesity is a major driver of inflammation^41^ and anti-inflammatory interventions may have a role in mitigating the adverse impacts of obesity on the systemic microvasculature. This may be a future therapeutic target given the recognition that the microvasculature is a key player in the obesity-associated CVD pathogenesis^42^. Currently, most interventions focus on nutritional intake and physical activity but do not result in sustained improvements in preventing or managing weight gain^43^. Addressing downstream adverse effects, such as inflammation, may be useful adjunctive interventions^44^. Further studies are required to understand the key therapeutic targets and the optimal age to intervene safely.

Our findings suggest that the association between obesity and wider retinal venules is mediated via inflammation, even at 11–12 years of age. Conversely, for retinal arterioles, non-inflammatory pathways appear more important.

## Methods

### Study design and participants

The Child Health CheckPoint (CheckPoint) is a cross-sectional population-based biophysical assessment substudy nested within the national Longitudinal Study of Australian Children (LSAC)^45,46^. Details of the initial study design and recruitment are outlined elsewhere^47,48^. Briefly, LSAC recruited a nationally representative birth cohort of 5107 infants using a 2-stage clustered design and has since collected data in biennial ‘waves’ for a decade. The initial response rate in 2004 was 57.2%, of whom 73.7% (n = 3764) were retained to wave 6 in 2014.

CheckPoint took place from February 2015 to March 2016, between LSAC waves 6 and 7 at child age 11–12 years. In total, 1874 children (53% of all wave 6 families) participated in this detailed cross-sectional biophysical assessment^46^. Most attended an assessment centre in seven cities around Australia. Families (n = 518) who had a more limited assessment in regional cities or home visits were not included as the equipment for retinal imaging could not be readily transported.

The CheckPoint study was approved by the Royal Children’s Hospital Melbourne Human Research Ethics Committee (33225D) and the Australian Institute of Family Studies Ethics Committee (14–26). The attending parents/caregivers provided written informed consent for themselves and their children to participate. All methods were performed in accordance with the relevant guidelines and regulations.

### Measures relating to obesity

Height and weight (to the nearest 0.1 cm and 0.1 kg respectively) were measured up to three times by trained assessors, where the mean of all measurements was used. BMI was calculated as weight (kg)/height (m^2^). Children’s BMI was converted into age- and gender-specific z-scores using the US Centres for Disease Control (CDC) growth reference charts^49^, and also classified as normal (5 to 85^th^ percentile), overweight (85^th^ to <95^th^ percentile) and obese (≥95^th^ percentile)^50^. For adults, the BMI status was defined as normal (18.5 to 24.9 kg/m^2^), overweight (25 to 29.9 kg/m^2^), or obese (≥30 kg/m^2^)^51^. We did not include participants with underweight given the limited number in this study (child BMI z-score <5th percentile, 4.2%; adults BMI < 18.5 kg/m^2^, 0.3%).

Waist circumference (cm) was measured against bare skin around the navel. Two measurements were taken and the mean was used. For children and adults, continuous WHtR was calculated as waist (cm)/height (cm) and also classified into two categories (<0.5 and ≥0.5), where a ratio ≥0.5 indicates central obesity^52,53^.

### Retinal vascular calibre

Two optic disc-centred digital photographs from each eye were taken by a fundus camera (EOS 60D SLR) in a darkened room without mydriasis. Experienced graders scored the images using the Integrative Vessel Analysis software program (IVAN, University of Wisconsin, Madison, USA). Details of scoring procedures are described elsewhere^54^. Briefly, right eye images were selected as the first choice for scoring. Retinal vessels were identified as arterioles or venules from a specific area (one-half to one-disc diameter from the optic disc margins) and a segment of each vessel within this area was selected for measurement. Diameters of all the selected segments were measured automatically using IVAN software. Summary estimates of the average retinal vascular calibre were calculated combining measurements of the six largest arterioles or venules^55^. Reproducibility of retinal vascular measurements has been reported, with inter- and intra-grader intraclass correlation coefficients of 0.76 to 0.99^54^.

### Inflammatory marker

At the assessment centre, semi-fasting (median 4.2 hours postprandial) peripheral blood was processed within four hours of collection at an on-site processing laboratory, with serum aliquots frozen at −80 °C. Serum samples were shipped on dry ice for high-throughput proton NMR spectroscopy (Nightingale Ltd, Vantaa, Finland). Levels of GlycA were calculated using Nightingale 2017 quantification algorithms and were reported in mmol/L^56^.

### Covariates

Age, sex and family socioeconomic position (SEP) were considered as potential confounders of the exposure-outcome, exposure-mediator and mediator-outcome associations^22,57,58^. SEP was measured in LSAC wave 6 (child age 10–11 years), approximately 12 months before the CheckPoint assessment. This SEP variable summarises parent-reported combined household income, current or most recent occupation of each parent, and highest educational qualification of each parent^59^. Each component was scaled and an unweighted average was calculated and standardised within the wave to have a mean of 0 and standard deviation (SD) of 1. A higher score indicates more advantaged socioeconomic position.

### Statistical methods

Statistical analyses were performed in R software (version 3.5.2). Data from children and adults were analysed separately, using the records with complete data on all the variables. Scores on all continuous exposure and outcome measures were internally standardised ([observed value-mean]/SD).

Firstly, we fitted linear regression models to explore associations of each exposure (BMI and WHtR as continuous variables) with the proposed mediator (GlycA) and with the outcome (retinal vascular calibre), as well as the association of the mediator with the outcome. Linear regression models were adjusted for age, sex and SEP. In addition, when examining associations between GlycA and retinal vascular calibre, models were additionally adjusted for BMI, as it is a potential confounder.

We then investigated the possible mediating role of GlycA using the causal mediation framework, while considering BMI and WHtR continuously and categorically (Fig. 1c), adjusted for age, sex and SEP^60^. Specifically, we estimated the so-called “interventional” direct and indirect effects, which are estimable under relaxed assumptions compared to previous causal mediation approaches^17,18^. The interventional indirect effect through M is interpreted as the change in the risk of the outcome Y in the exposed if we were to shift the distribution of M to that in the unexposed, and the interventional direct effect is the effect of X on Y that would remain after that shift. We used the “mediation” R package^61^, and specified the X, Y, and M in the model as continuous and categorical BMI/WHtR, retinal arteriolar/venular calibre and GlycA (Fig. 1c). This utilises a g-computation approach with linear regression for the outcome and mediator given their antecedent variables as building blocks. The direct and indirect effect estimates indicate the magnitude of change (in SDs) in the outcome variable per SD change in the continuous exposure variable or compared to the categorical reference group (i.e. normal-weight BMI and <0.5 WHtR category).

## Supplementary information

Supplementary information.

## Data Availability

The datasets generated during and/or analysed during the current study are available from the corresponding author on reasonable request.
