# Supplementary material for: Inflammation mediates the relationship between obesity and retinal vascular calibre in 11-12 year-olds children and mid-life adults
Source: Sci Rep. 2020 Mar 19;10:5006. doi: 10.1038/s41598-020-61801-w (PMC7081237; doi:10.1038/s41598-020-61801-w)
Supplement: Supplementary file 1 — Supplementary information. [file 41598_2020_61801_MOESM1_ESM.pdf]

**Title: Inflammation mediates the relationship between obesity and retinal vascular calibre in 11-12 year-olds children and mid-life adults**

**Authors:**

Mengjiao Liu, Kate Lycett, Margarita Moreno-Betancur, Tien Yin Wong, Mingguang He, Richard Saffery, Markus Juonala, Jessica A. Kerr, Melissa Wake, David P. Burgner

**Supplementary figure.** Participant flowchart

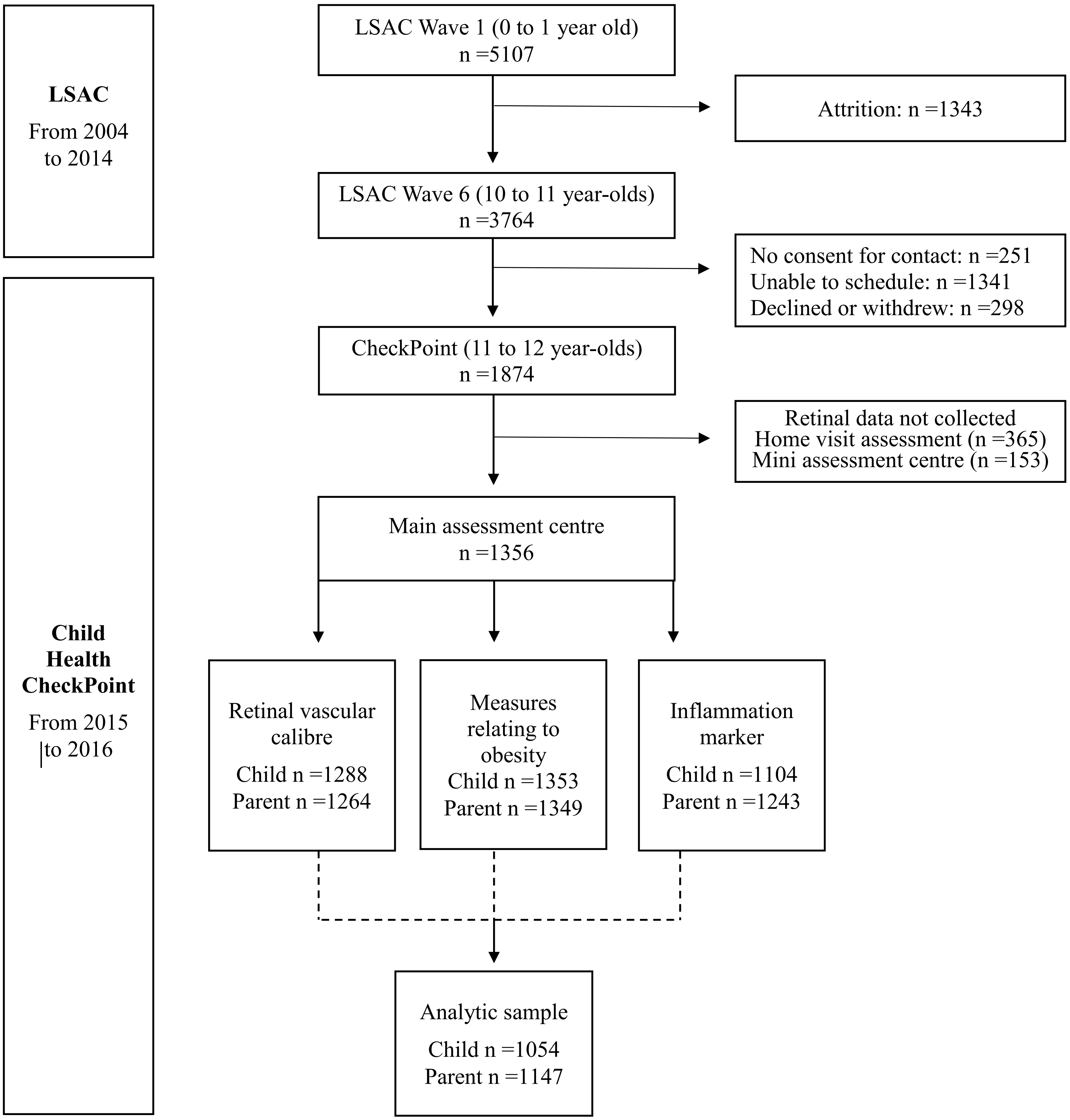


N: number of responses; LSAC, Longitudinal Study of Australian Children

**Supplementary table.** Associations of GlycA with retinal vascular calibre with adjustment for age, sex, SEP, BMI and LDL.

| | Children | | Adults | |
|---|---|---|---|---|
| **Outcome measures** | **Effect size (95% CI)** | ***p*** | **Effect size (95% CI)** | ***p*** |
| Retinal arteriolar calibre | 0.004 (-0.06, 0.07) | 0.29 | -0.03 (-0.10, 0.05) | 0.49 |
| Retinal venular calibre | 0.07 (0.01, 0.14) | 0.03 | 0.03 (-0.04, 0.11) | 0.37 |
